# Temporal and spatial control of transgene expression using laser induction of the *hsp70 *promoter

**DOI:** 10.1186/1471-213X-6-55

**Published:** 2006-11-20

**Authors:** Diane M Ramos, Firdous Kamal, Ernst A Wimmer, Alexander N Cartwright, Antónia Monteiro

**Affiliations:** 1 Department of Biological Sciences, University at Buffalo, Buffalo, NY 14260, USA; 2 Department of Electrical Engineering, University at Buffalo, Buffalo, NY 14260, USA; 3 Department of Developmental Biology, Johann-Friedrich-Blumenbach-Institute of Zoology and Anthropology, Georg-August-University Göttingen, GZMB, 37077 Göttingen, Germany; 4 Department of Ecology and Evolutionary Biology, Yale University, New Haven, CT 06511, USA

## Abstract

**Background:**

Precise temporal and spatial regulation of transgene expression is a critical tool to investigate gene function in developing organisms. The most commonly used technique to achieve tight control of transgene expression, however, requires the use of specific DNA enhancers that are difficult to characterize in non-model organisms. Here, we sought to eliminate the need for this type of sequence-based gene regulation and to open the field of functional genetics to a broader range of organisms.

**Results:**

We have developed a new laser mediated method to heat shock groups of cells that provides precise spatio-temporal control of gene expression without requiring knowledge of specific enhancer sequences. We tested our laser-system in a transgenic line of *Bicyclus anynana *butterflies containing the *EGFP *reporter gene attached to the heat sensitive *hsp70 *promoter of *Drosophila melanogaster*. Whole organismal heat shocks demonstrated that this *Drosophila *promoter can drive gene expression in butterflies, and the subsequent laser heat shocks showed that it was possible to activate cell-specific gene expression in very precise patterns on developing pupal wings.

**Conclusion:**

This laser-mediated gene expression system will enable functional genetic investigations, i.e., the ectopic expression of genes and their knock-down in targeted groups of cells in model and non-model organisms with little or no available regulatory data, as long as a compatible heat-shock promoter is used and the target tissue is accessible to a laser beam. This technique will also be useful in evolutionary developmental biology as it will enable the study of the evolution of gene function across a variety of organisms.

## Background

The use of transgenic animals has helped to produce major advances in the field of functional genetics. Typically, these experiments use only model organisms with known regulatory DNA sequences, i.e. enhancers, that drive gene expression at particular times in development and in particular cells. But, while transposable elements such as piggyBac have enabled transgenic manipulations of increasingly diverse organisms[1], the lack of versatile tools for genetic manipulations in these organisms has hindered their use in functional genetic experiments. Specifically, there is a need to provide temporal and spatial control of transgene expression because it is known that within a developmental context, a gene expressed ubiquitously produces different effects than the same gene expressed in a more restricted pattern [2].

In model organisms, dramatic genetic manipulations utilizing the widely used yeast GAL4/UAS system have increased our understanding of gene function [3]. As the field of functional genomics moves toward a more comparative framework however, the GAL4/UAS system has certain drawbacks that might limit its usefulness. Traditionally, researchers must create extensive transgenic lines to bring the GAL4 transcription factor under the control of an appropriate regulatory sequence. This is both time and labor intensive and requires maintenance of a large number of animal stocks. Modifications to the traditional GAL4/UAS system can eliminate the requirement for specific GAL4 lines but do not reproduce the precise spatial control of transgene expression[4].

Inducible promoters are also utilized to misexpress genes in transgenic organisms. The *hsp70 *promoter is a popular inducible promoter in a variety of systems including invertebrates such as *Drosophila*[5,6] and *Bombyx mori*[7]. While whole organismal heat stress is the most commonly used induction method for this promoter, researchers have found novel ways to provide temporal and spatial control of induction including heated needles[8,9] and laser systems. In particular, micro laser pulses of a modified cell ablation system were used to heat shock single cells and induce the *hsp70 *promoter in *C. elegans*[10], *Drosophila*[11] and *Danio rerio*[12]. Our study represents the first application of laser-mediated promoter induction to larger populations of cells, providing the fine level of spatial control necessary to replicate many of the elaborate expression patterns observed during development.

## Results and Discussion

**Figure 1:**
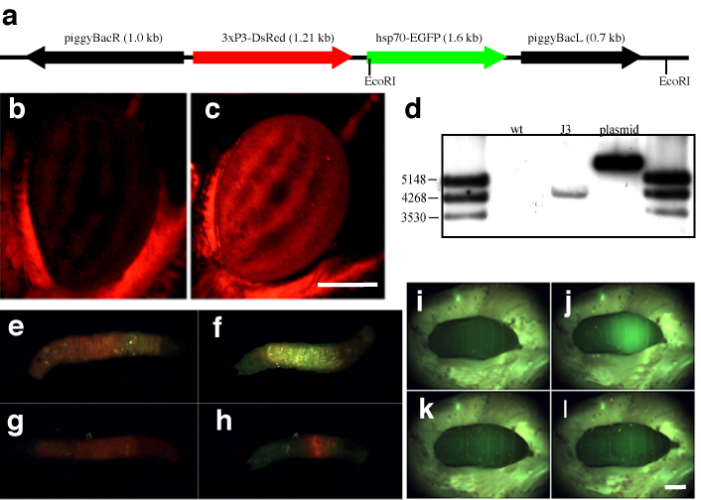
**Creating and testing the transgenic line of butterflies**. **(a) Plasmid illustration of piggyBac [3xP3-DsRed, hsp70-EGFP] (b) **Eye of a wild-type *B. anynana *adult versus **(c) **eye of a transgenic individual showing DsRed expression. Scale bar = 500 μm. (d) Southern blot of *Eco*RI digests of wild-type (wt) genomic DNA, J3 transgenic line genomic DNA and *pBac{3xP3-DsRed, Hsp70-EGFP} *plasmid. Blot was probed for *DsRed *PCR fragment. J3 transgenic line is carrying a single insertion of the piggyBac element. **(e, f) **Transgenic first instar larva before and after heat shock. **(g, h) **Wild-type first instar larva before and after heat shock. **(i, j) **Transgenic pupa before and after heat shock. **(k, l) **Wild-type pupa before and after heat shock. Scale bar = 2 mm.

In order to develop this *hsp70*-laser induction method, we generated a *piggyBac *construct that carried the *Drosophila hsp70 *promoter driving the reporter gene *EGFP *as well as the synthetic *3xP3 *promoter driving *DsRed *expression in the eyes as a transformation marker (Fig. 1a). This construct was then used for germ-line transformations of *Bicyclus *as previously reported[13]. F_1 _individuals were screened for DsRed fluorescence in the adult eye (Fig. 1b,c). Six putative transgenic individuals were isolated from separate populations and four were confirmed by PCR. The transformation rate was 4.2%, which is similar to previous reports in Lepidoptera [13,14]. One line (J3) was established for use in all the heat shock experiments. Southern Blot characterization showed the J3 line carried a single insertion of the *piggyBac *element (Fig. 1d). Every generation, individuals were selected either for *DsRed *expression, or *EGFP *expression after a 1 h heat-shock at 39°C, in the eyes. Initially, screens were also confirmed by PCR targeting the *EGFP *gene using larval hemolymph samples[15].

We tested the function of the *Drosophila hsp70 *promoter in *Bicyclus anynana *using whole organismal heat shocks at several stages of development. Animals were placed in a 39°C chamber for 1 to 2 hours. The animals were then moved to a 27°C chamber for at least 4 hours prior to visualization. EGFP was detectable 4 to 24 hours after the onset of the heat shock. In larval samples, EGFP was visible in the epidermis (Fig. 1e–h) and in the gut (data not shown). In pupal samples, EGFP was mostly visible in the dorsal region of the abdomen (Fig. 1i–l) but was visible in the wing epidermis upon dissection (data not shown).

**Figure 2:**
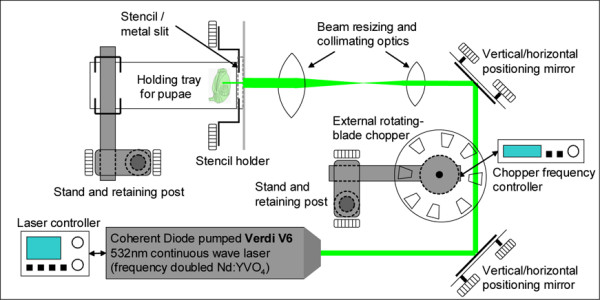
Schematic of laser setup used for the heat shocks.

**Figure 3:**
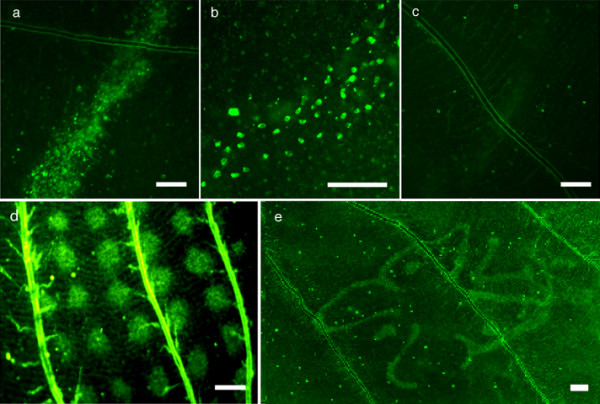
**Laser mediated heat shocks**. **(a) **Transgenic wing tissue showing EGFP expressing cells as a result of a line heat shock. **(b) **Higher magnification of EGFP expressing cells. **(c) **Wildtype wing tissue after line heat shock. **(d) **Complex grid pattern of EGFP expression. **(e) **Complex butterfly pattern of EGFP expression as a result of laser heat shock. Scale bar = 100 μm in all panels.

We then tested the ability of a laser (Fig. 2) to induce *hsp70*-*EGFP *expression in a simple line pattern in the epidermal cells of the pupal wing of transgenic butterflies (n = 45) and wildtype controls (n = 27). We observed green fluorescence in a subset of epidermal cells that corresponded to the treated area in 75.5% of J3 individuals (n = 34; Fig. 3a,b). Given that the J3 line is not a pure homozygous line and that wild-type individuals are still segregating in the population, we believe this percentage underestimates the actual percentage of positive results in transgenic individuals. While most of the wild-type control animals showed no pattern of fluorescence (85%, n = 23; Fig. 3c), in 4 animals we observed some green autofluorescence from damaged cells. Using a dye that reacts within membrane compromised cells to produce a blue fluorescent signal, we confirmed that the observed green fluorescence in treated J3 individuals was due to the presence of EGFP (data not shown). To eliminate the problem of autofluorescence in the green channel, laser-treated wings from both J3 and wild-type individuals were stained with a rabbit anti-EGFP primary antibody and a Texas-Red conjugated anti-rabbit secondary antibody to shift the EGFP signal to the red channel. In J3 individuals, a red line was observed that corresponded to the treated area while no signal was detected in wild-type individuals (data not shown).

To determine the effect of the laser treatment on adult wings, we treated 12 transgenic and 12 wild-type individuals and allowed the adults to eclose. We then compared the wings of treated individuals to those of untreated controls. No pattern or vein disruptions were observed in any of the treated individuals.

In order to determine the spatial resolution of our laser system, we heat shocked cells in a variety of complex patterns produced using metal micro-stencils. For these experiments, the stencils replaced the metal slit in the center of the beam path (Fig. 2). The average intensities were comparable to those of the previous experiment. We found that the laser treatment can produce complex patterns of EGFP induction in the pupal wing (Fig. 3d,e), in distinct populations of cells separated by as few as 20 cell diameters (Fig. 3d).

Given the tight control of gene expression possible with this system, we intend to utilize it to provide some of the first functional tests of the roles of candidate genes in the development of wing patterns in *B. anynana*. We do not feel that this system is restricted to this use and could easily be adapted to investigate gene function in a number of organisms and tissues including developing plant meristems, or epidermal stem cells in vertebrates. The wavelength we used in these initial experiments do produce some damage and cell death after 20 minutes of heat-shock, therefore, we are currently testing a new laser that uses lower energy wavelengths in the infrared spectrum.

## Conclusion

We have shown that laser-mediated heat shocks can be used to induce the *Drosophila hsp70 *promoter in groups of cells of the pupal wings of *B. anynana*. This technique will be useful to study gene function in other biological systems where transgenesis is possible but data on regulatory sequences is lacking. Our method will allow researchers to test candidate genes in any tissue accessible to laser treatment with only the need of a compatible heat-shock promoter. The spatial and temporal control offered by this system is a powerful new tool to investigate various roles of a candidate gene's function in the ontogeny of an organism. In our research organism, *B. anynana*, the laser treatment did not alter the pattern of the adult wing allowing us to use this technique to test the function of candidate genes in color patterning the butterfly wing.

## Methods

### Plasmid construction

*pBac{3xP3-DsRed, hsp70-EGFP}: pSLfaHSfa*, a general shuttle vector for cloning heat shock constructs, was created by inserting the 0.9 kb *Xho*I(blunted)-*Hin*dIII fragment of pCaSpeR-hs[16], that contains a *Drosophila *heat shock cassette, into *Eco*RI-(blunted) and *Hin*dIII-opened *pSLfa1180fa*[17]. Into this vector opened with *Bgl*II and *Not*I, the 0.8 kb *Bam*HI-*Not*I fragment of *pSL-*EGFP [18] was inserted to generate *pSLfaHS-EGFPfa*. The heat shock inducible *EGFP *cassette was then taken from there as a 1.7 kb *Asc*I fragment and cloned into *Asc*I-opened *pBac{3xP3-DsRedaf}*[19] in a way that both the marker *3xP3-DsRed *and *Hsp70-EGFP *are transcribed in the same orientation (Fig 1a).

### Transgenesis

*pBac{3xP3-DsRed, hsp70-EGFP} *and helper plasmid (*pHsp82Pbac *[20]) in equal concentrations of 246 ng/μl were injected into 4009 eggs within 1 hour post-oviposition. 277 larvae emerged from the injected eggs resulting in 143 adults. Adults were sexed and separated within 24 hours of eclosion. Groups of 10 injected individuals of the same sex were backcrossed to wild-type of the opposite sex in a ratio of 1:2. F_1 _individuals were screened for DsRed fluorescence in the adult eyes using a Nikon SMZ1500 fluorescent microscope. Selected F_1 _adults were backcrossed to wild-type in single pair cages. After 1–2 weeks of egg laying, F_1 _adults were sacrificed and genomic DNA was extracted using a DNAeasy kit (Qiagen). In subsequent generations, we performed brother and sister matings within each line, selecting only animals with the brightest eyes in order to increase the frequency of the transgene. We kept one line, the J3 line, for subsequent molecular characterization and laser experimentation.

### PCR screen for EGFP

All PCR screens used (5'-CGTGACCACCTTGACCTAC-3') and (5'-TGATCGCGCTTCTCGTT-3') forward and reverse primers respectively. PCR cycling conditions were 1 × 94°C, 2 min; 40 × (94°C, 30 s; 56.5°C, 30 s; 72°C, 30 s); 1 × 72°C, 10 min.

### Southern Blot

A probe for *DsRed *was made by PCR using the *pBac{3xP3-DsRed, hsp70-EGFP} *plasmid as template, (5'-GGTGCGCTCCTCCAAGAAC-3') and (5'-TGCGCTCGTACTGCTCCAC-3') forward and reverse primers respectively and a DIG DNA labeling mix (Roche, 11277065910). PCR cycling conditions were 1 × 94°C, 2 min; 35 × (94°C, 30 s; 60°C, 30 s; 72°C, 30 s); 1 × 72°C, 6 min. A dot blot test of the probe sensitivity produced a strong signal with 510 pg of template plasmid. Genomic DNA was extracted from single individuals as previously described[21] with an additional 1:1 phenol:chloroform purification. 68 μg and 66.5 μg of J3 and wt gDNA, respectively, were digested overnight with 5U/ug *Eco*RI. Digestions were cleaned using 1:1 phenol:chloroform and gDNA was precipitated in ethanol overnight. 42 μg of J3 and wt gDNA was loaded on gel along with DIG labeled size markers (Roche, 11218603910) and *pBac{3xP3-DsRed, hsp70-EGFP} *plasmid. Following an overnight gel run, the DNA was transferred to a Hybond N+ membrane, which was subsequently hybridized overnight to a *DsRed *probe at 55°C. In the morning, the membrane was incubated with an alkaline phosphatase conjugated anti-DIG antibody (1:10,000 dilution, Roche, 11755633001). The alkaline phosphatase substrate CSPD (Roche, 11755633001) was used to produce a chemi-luminescent signal that was detected by exposure to an X-ray film.

### Laser Heat-shocking treatments

Using two biconvex lenses (fl = 50 mm and 100 mm) the output beam of a Coherent Diode pumped Verdi V6 solid state 532 nm continuous wave laser was collimated to a diameter of 2.41 mm (Fig. 2). A metal slit (180 μm × 1.39 mm) was placed at the center of this beam path, whereas metal micro-stencils (Metal Etching Technology Associates Inc., Lumberton, NJ) were used for a subset of these experiments. Pupae were positioned such that the narrow laser line illuminated cells along the anterior posterior axis of the wing. Power measured through this slit was 25 mW. Average intensities were 10 W/cm^2^. Beam widths were measured as 10% to 90% of full power using a linear translator and a Thorlabs LM-2 optical power meter. We found that it was necessary to use an external chopper (rotating blade with equally spaced openings from Stanford Research Instruments; frequency set to 5 Hz) to modulate the beam in order to prevent heat dispersion into nearby regions and to obtain a greater spatial control of gene expression. Over the course of our experiments, we observed that pupal age was a critical variable for the success of the heat shock treatment. The parameters presented here are optimized for pupae 16–20 hours post-pupation. Heat shocks were 17–20 minutes in duration. The wings were dissected from the pupae 12–16 hours after laser treatment in order to allow EGFP to be produced, and visualized on a Leica DMIRE2 fluorescent compound microscope. In other systems, parameters to optimize include the duration of the heat-shock, the frequency with which the chopper interrupts the laser, and the overall power of the laser.

### Live-Dead staining

Pupal wings were extracted and incubated for 30 minutes on ice in wells containing 0.5 μl of a blue-fluorescent reactive dye (Catalog # L-23105, Molecular Probes, Eugene, Or). Wings were mounted in 90% glycerol/100 mM Tris pH 8.0.

### Anti-GFP staining

Pupal wings were extracted and stained as previously described [22] using a rabbit anti-GFP primary antibody (1:200 dilution, Molecular Probes, A-11122) and Texas-Red anti-rabbit secondary antibody (1:200 dilution, Molecular Probes, T-2767). Wings were mounted in SlowFade mounting medium (Molecular Probes, S-7461).

## Authors' contributions

DMR, FK and AM collected the data in this study, EAW produced the transgenic construct, ANC supervised the laser design, AM and EAW conceptualized the study, and DMR wrote the manuscript with help from the other authors. All authors read andapproved the final manuscript.
